# Positive Darwinian selection is a driving force for the diversification of terpenoid biosynthesis in the genus *Oryza*

**DOI:** 10.1186/s12870-014-0239-x

**Published:** 2014-09-16

**Authors:** Hao Chen, Guanglin Li, Tobias G Köllner, Qidong Jia, Jonathan Gershenzon, Feng Chen

**Affiliations:** Department of Plant Sciences, University of Tennessee, Knoxville, TN 37996 USA; Key Laboratory of Plant Resources Conservation and Sustainable Utilization, South China Botanical Garden, Chinese Academy of Sciences, Guangzhou, 510650 China; College of Life Sciences, Shaanxi Normal University, Xi’an, 710062 China; Max Planck Institute for Chemical Ecology, Hans-Knöll-Strasse 8, D-07745 Jena, Germany; Graduate School of Genome Science and Technology, University of Tennessee, Knoxville, TN 37996 USA

**Keywords:** Plant secondary metabolism, Terpene synthase, Positive selection

## Abstract

**Background:**

Terpenoids constitute the largest class of secondary metabolites made by plants and display vast chemical diversity among and within species. Terpene synthases (TPSs) are the pivotal enzymes for terpenoid biosynthesis that create the basic carbon skeletons of this class. Functional divergence of paralogous and orthologous *TPS* genes is a major mechanism for the diversification of terpenoid biosynthesis. However, little is known about the evolutionary forces that have shaped the evolution of plant *TPS* genes leading to terpenoid diversity.

**Results:**

The orthologs of *Oryza Terpene Synthase 1* (*OryzaTPS1*), a rice terpene synthase gene involved in indirect defense against insects in *Oryza sativa*, were cloned from six additional *Oryza* species. *In vitro* biochemical analysis showed that the enzymes encoded by these *OryzaTPS1* genes functioned either as (*E*)-β-caryophyllene synthases (ECS), or (*E*)-β-caryophyllene & germacrene A synthases (EGS), or germacrene D & germacrene A synthases (DAS). Because the orthologs of OryzaTPS1 in maize and sorghum function as ECS, the ECS activity was inferred to be ancestral. Molecular evolutionary detected the signature of positive Darwinian selection in five codon substitutions in the evolution from ECS to DAS. Homology-based structure modeling and the biochemical analysis of laboratory-generated protein variants validated the contribution of the five positively selected sites to functional divergence of OryzaTPS1. The changes in the *in vitro* product spectra of OryzaTPS1 proteins also correlated closely to the changes in *in vivo* blends of volatile terpenes released from insect-damaged rice plants.

**Conclusions:**

In this study, we found that positive Darwinian selection is a driving force for the functional divergence of OryzaTPS1. This finding suggests that the diverged sesquiterpene blend produced by the *Oryza* species containing *DAS* may be adaptive, likely in the attraction of the natural enemies of insect herbivores.

**Electronic supplementary material:**

The online version of this article (doi:10.1186/s12870-014-0239-x) contains supplementary material, which is available to authorized users.

## Background

Plants produce diverse secondary metabolites that are not essential for growth and development but play important roles in plant interactions with other organisms [1,2]. With over 25,000 representatives [3], terpenoids constitute the largest class of plant secondary metabolites [4,5]. Synthesized as the components of resins, complex oils, or volatile mixtures (such as floral scents) [6], terpenoids are involved in diverse biological processes ranging from plant defense to reproduction and symbiosis [4,5,7]. Despite the collective diversity of terpenoids in the plant kingdom, for any given plant species, only a subset of terpenoids are produced, some of which may be unique to the taxon [8,9]. Such taxon-specific diversity may be important for the specific biological functions of terpenoids. Therefore, understanding the evolutionary mechanisms underlying the diversification of terpenoid biosynthesis is important for us to understand plant adaptation to specialized niches.

The wealth of structural diversity of plant terpenoids can be mainly attributed to an enzyme class known as terpene synthases (TPSs). TPSs convert the isoprenyl diphosphate precursors geranyl diphosphate (GPP), farnesyl diphosphate (FPP) and geranylgeranyl diphosphate (GGPP) to a multitude of cyclic and acyclic monoterpenes (C10), sesquiterpenes (C15) and diterpenes (C20), respectively [5,10]. The unusual reaction mechanism of TPSs involves the formation of intermediate carbocations that can have multiple metabolic fates, depending on the termination mechanisms [10,11]. As a result, a single TPS can catalyze the formation of multiple terpene products from a single substrate. Therefore, the enormous diversity of plant terpenoids is partly due to the ability of some plant TPSs producing multiple products.

The principal cause of plant terpenoid diversity is the large number of TPSs with different product specificities. In most sequenced plant genomes that have been analyzed, *TPSs* constitute mid-sized gene families with 30–100 members which are most likely to have evolved through gene duplication followed by functional divergence. These genes can be further divided into subfamilies based on their evolutionary relatedness with individual families generally associated with the formation of a specific class of terpenoids, such as monoterpenes, sesquiterpenes or diterpenes [12]. The individual members in specific TPS subfamilies usually share a specific type of isoprenyl diphosphate as substrate, but exhibit large variations in product profiles [12]. The biochemical basis for such functional divergence has been studied using biochemical and structural approaches. The product profiles of many TPSs have been interconverted by mutating only a small number of amino acid residues within or around the active site cavity [13-16]. On the other hand, numerous remote substitutions that result in the repositioning of key residues within the active site cavity can lead to enzymes with moderate level of sequence homology catalyzing similar biochemical reactions [17]. In addition to individual sites, epistasis, which is defined as context dependence for mutational effects, also plays a critical role on product specificity of terpene synthases [18]. Other mechanisms such as losing the N-terminal domain [19] or fusing two functional domains [20] have been proposed to be involved in evolving new terpene synthase genes.

In contrast, our knowledge of the molecular evolutionary basis underlying functional divergence of TPSs is very limited. The evolution of the large TPS gene family probably occurred largely through gene duplication followed by functional divergence, but the driving forces behind this process have not been well investigated. In contrast, positive Darwinian selection has been identified to be an important driving force for the evolution of a number of genes of plant secondary metabolism for functional diversification. Such examples include the methylthioalkylmalate synthases involved in the glucosinolate biosynthesis [21], the shikimate kinase (SK) of the shikimate pathway [22], the methyltransferases of the SABATH family important for the production of methyl esters [23], the dihydroflavonol-4-reductase (DFR) involved in anthocyanin biosynthesis [24] and the homospermidine synthase involved in pyrrolizidine alkaloid biosynthesis [25]. While it may be sensible to propose that positive selection has also played a role in the functional diversification of TPSs, no such evidence has been presented.

We have chosen rice (*Oryza*) as a model plant to study the molecular evolution of *TPS* genes for two main reasons. First, rice plants produce a mixture of approximately 20 volatile monoterpenes and sesquiterpenes with a clear biological function. Produced upon herbivore damage, they serve in attracting the natural enemies of herbivores [26]. Second, the molecular basis of volatile terpene production in rice has been well characterized. Three *TPS* genes *Os02g02930*, *Os08g07100* and *Os08g04500* are responsible for production of the majority of the terpenes released from insect-damaged rice plants [26]. This study focused on the sesquiterpene synthase Os08g04500 from *O. sativa* and its orthologs from selected *Oryza* species. Os08g04500 produces predominantly (*E*)-β-caryophyllene and germacrene A [26]. The orthologs of Os08g04500 in maize (TPS23) [27] and sorghum (SbTPS4) [28] which also play a role in attracting herbivore enemies produce only (*E*)-β-caryophyllene as their major product. The common ancestry of these three grasses indicates that functional divergence of the Os08g04500/TPS23/SbTPS4 orthologs has occurred, and such functional changes may have happened within the *Oryza* genus as well. Biochemical characterization and molecular evolutionary analysis of the orthologs of Os08g04500 (collectively designated as OryzaTPS1) from multiple *Oryza* species implied that positive Darwinian selection is one evolutionary force driving the functional divergence of *OryzaTPS1s*.

## Results

### Functional conservation and divergence of OryzaTPS1s

To detect sequence divergence of *OryzaTPS1* in rice, *OryzaTPS1s* were cloned from six additional *Oryza* species including *O. glaberrima* (African cultivated rice), *O. rufipogon* (a perennial wild relative of *O. sativa*), *O. nivara* (an annual wild relative of *O. sativa*), *O. barthii* (a wild relative of *O. glaberrima*), *O. glumaepatula* and *O. officinalis* (Additional file 1). All these seven species are diploids. The seven *OryzaTPS1s* cloned from the seven *Oryza* species displayed 96-99% similarity at the protein sequence level (Additional file 2). The related sequences have been deposited in the GenBank under accessions KJ415250 to KJ415255.

**Figure 1:**
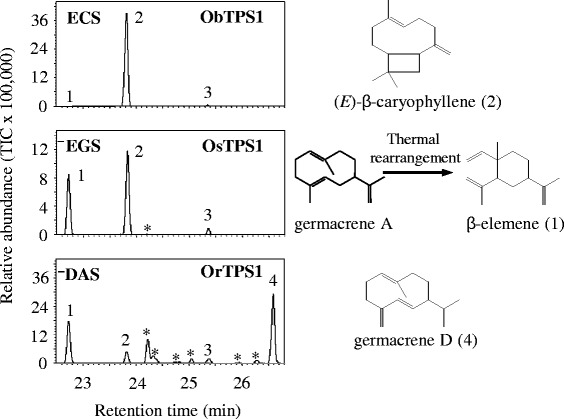
**OryzaTPS1s have diverged to produce three different sesquiterpene product spectra.** The enzymes were expressed in *E. coli*, extracted, and incubated with the substrate (*E*, *E*)-farnesyl diphosphate. The resulting terpene products were separated by gas chromatography–mass spectrometry (GC-MS). The traces of the MS detector were shown for ObTPS1, a representative (*E*)-β-caryophyllene synthase (ECS), for OsTPS1, a representative (*E*)-β-caryophyllene/germacrene A synthase (EGS), and for OrTPS1, the germacrene D & germacrene A synthase (DAS). Products were identified as 1, β-elemene; 2, (*E*)-β-caryophyllene; 3, α-humulene; 4, germacrene D by comparison of their retention times and mass spectra to those of authentic standards. *Unidentified sesquiterpenoids. The chemical structures of compounds 1, 2 and 4 were shown on the right. Note: β-elemene was produced as a thermal rearrangement product from germacrene A in the GC injector (Additional files 4 and 5).

Our previous study showed that OryzaTPS1 in *O. sativa* Nipponbare (Os08g04500, renamed as OsTPS1 here) functions as a sesquiterpene synthase catalyzing the formation of multiple sesquiterpenes, with (*E*)-β-caryophyllene as the major product and germacrene A as the next most abundant [26]. Here it was designated as an (*E*)-β-caryophyllene/germacrene A synthase (EGS). For functional evaluation, the six *OryzaTPS1s* other than OsTPS1 were expressed in *E. coli* and recombinant proteins were tested for terpene synthase activity. All these proteins were biochemically active. These enzymes fell into three categories based on their biochemical activities (Figure 1 and Additional files 3, 4 and 5). Two of them functioned as EGSs, while three others produced (*E*)-β-caryophyllene as the only dominant product with α-humulene, germacrene A and occasionally germacrene D as minor products. These were designated as (*E*)-β-caryophyllene synthase (ECS). One other enzyme, OrTPS1, produced germacrenes D and A as major products and was therefore designated as germacrene D & germacrene A synthase (DAS). In addition, as described in our previous reports [27,28], TPS23 and SbTPS4, the orthologs of OryzaTPS1 in maize and sorghum, are both ECSs. The ancestor of these orthologs occurred before the split of rice (*Oryza*), maize and sorghum as revealed by collinearity and phylogenetic analysis [28]. Thus, it is most likely that the ECS activity evolved before this split and served as the ancestral activity for the OryzaTPS1s of various *Oryza* species to diverge after the spilt. The fact that germacrene A and germacrene D were already minor products of ECS catalysis further supports that the sesquiterpene profiles of EGS and DAS evolved from a certain ancestral ECS.

### Molecular evolutionary analysis of *OryzaTPS1s*

**Figure 2:**
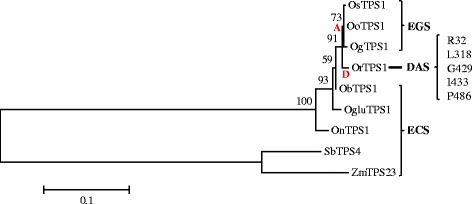
**Phylogenetic and evolutionary analysis of** ***OryzaTPS1s*** **.** The phylogenetic tree of *OryzaTPS1s* was constructed using the maximum likelihood method. All evolutionary analyses with the codon models were conducted using unrooted topologies. Based on the biochemical function of *OryzaTPS1s*, evolution occurred in two lineages, labeled as A (for germacrene A) and D (for germacrene D). Branch labels were bootstrap values based on 1000 replicates. Five sites of the germacrene D lineage where positive selection was detected were depicted on the right of the tree.

To further explore the evolution of OryzaTPS1s, its phylogeny was reconstructed. As shown in Figure 2, a striking feature of this gene tree was the strict clustering of the seven *OryzaTPS1* sequences for each of the three different biochemical activities. Several codon-based models implemented in the PAML package [29] were employed to analyze the evolutionary patterns reflected in this phylogeny. The one-ratio branch model indicated overall purifying selection for *OryzaTPS1* evolution (d*N*/d*S* (ω) = 0.63, Table 1). However, as selective pressure may diverge along different lineages [29], we applied the two-ratio models to examine whether positive selection can be detected in lineages A and D (Figure 2) where the original enzyme functional evolution may have occurred. Positive selection was detected in lineage D (ω = 999.0000, P < 0.05) but not in lineage A (Table 1). Failure to detect positive sites in lineage A may be due to the involvement of few substitutions which cannot be detected after the selective pressure was averaged on the branch. Since positive selection typically acts on only a few sites [30], we then used the branch-site models to examine whether there were signatures for selection at individual sites along lineage D. Likelihood ratio tests showed evidence for significant positive selection in lineage D (P < 0.05, Table 1). Five residues were identified to be under positive selection, including residues 32, 318, 429, 433, and 486 (using the OrTPS1 sequence as a reference, posterior probabilities > 95%, Figure 2 and Table 1). As the gene tree of *OryzaTPS1* was different from the canonical *Oryza* species tree [31], we also subjected the *OryzaTPS1* species tree to the same analysis. This analysis confirmed the outcome of the analysis using the gene tree (Additional file 6). Taken together, these results suggest that at least the evolution from ECS to DAS is adaptive.

**Table 1:** **Molecular evolutionary analysis of** ***OryzaTPS1s***

**Model**	**Parameters estimated**					**LnL**	**Significant?**	**Positively selected sites** ^**a**^
***Branch models***								
1ω	ω_0_ = 0.6296					−2775.1401		
2ω_D_	ω_0_ = 0.5532; ω_D_ = 999.0000					−2772.6923	Yes (P < 0.05)	
***Branch-site models***								
Null (M1a)	ω_0_ = 0; ω_1_ = 1					−2878.4062		
Null (neutral)	Site class	0	1	2a	2b	−2772.1400		
	ω_B_^b^	0	1	0	1			
	ω_A_	0	1	0.4000	0.4000			
Positive selection	Site class	0	1	2a	2b	−2769.7561	Yes (vs neutral) (P < 0.05)	32R (0.977*)
								318 L (0.976*)
	ω_B_^b^	0	1	0	1			429G (0.978*)
	ω_A_	0	1	179.3823	179.3823			433I (0.955*)
								486P (0.977*)

^a^The amino acids and their positions refer to those of OrTPS1.

^b^For Background.

*Denotes posterior probabilities by Bayes Empirical Bayes analysis.

### Functional validation of the five positively selected sites

**Figure 3:**
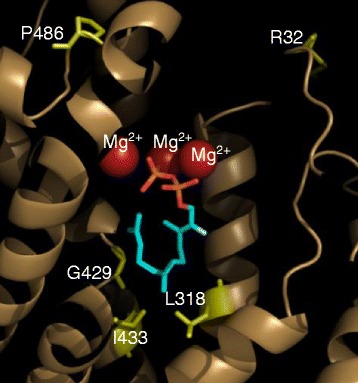
**A structural model of OrTPS1.** The homology-based model was created using the crystal structure of the tobacco 5-*epi*-aristolochene synthase M4 mutant ([32], complexed with (2-trans,6-trans)-2-fluorofarnesyl diphosphate) as template. The locations of the three magnesium ions and the substrate analogue were adopted from the modeling template.

Next we tested the role of the five sites at which positive selection was indicated in functional evolution from an (*E*)-β-caryophyllene-predominating activity to a germacrene D-predominating activity. First, a structural model of OrTPS1 (a DAS) was created based on its homology to the known structure of tobacco 5-*epi*-aristolochene synthase [32]. The residues 318, 429, 433 of OrTPS1 were located in the active site cavity, while residues 32 and 486 were positioned near the entrance of the active site cavity (Figure 3), providing initial evidence for the importance of all five sites in the functional evolution of OryzaTPS1.

**Figure 4:**
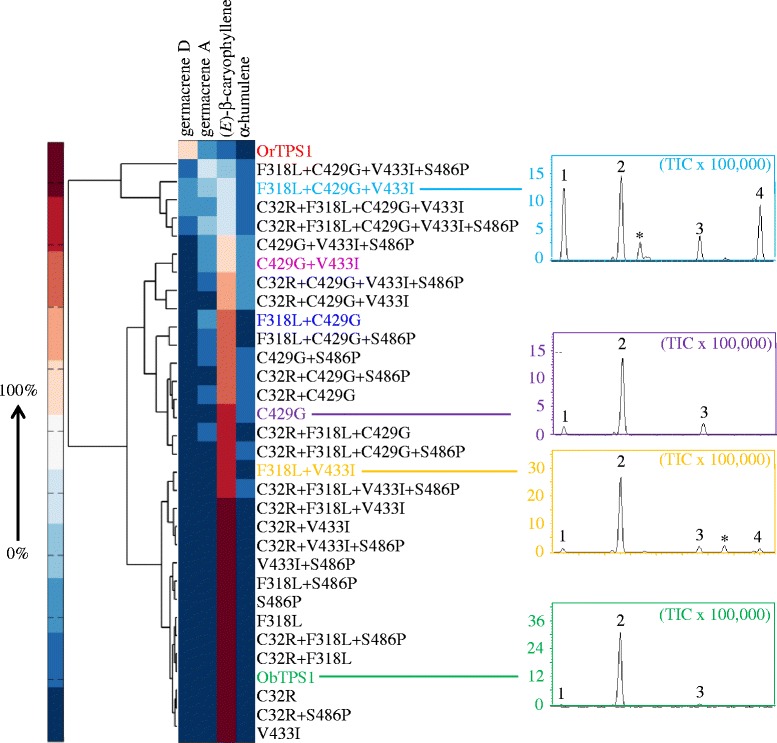
**The sites of positive selection in OryzaTPS1 helped to determine which sesquiterpene products were formed.** Hierarchical cluster analysis was performed for ObTPS1, OrTPS1 and the 31 ObTPS1 mutants. For each enzyme, the relative abundance of each sesquiterpene product was expressed as a percentage of the total amount of the four products. The color range represented percentages from 0% (a target compound was not detected at the expected retention time) to 100% abundance. Average values from three replicates were used. On the right, simplified chromatograms were depicted showing the relative abundance of the products of ObTPS1 (representing the ancestral (*E*)-β-caryophyllene synthesizing activity, ECS), ObTPS1-C429G (the only single mutant producing more germacrene A than ObTPS1), ObTPS1-F318 + V433I (the typical double mutant producing detectable germacrene D) and ObTPS1-F318 + C429G + V433I (the only triple mutant producing comparable amounts of germacrene A, germacrene D and (*E*)-β-caryophyllene). Products were identified as 1, β-elemene; 2, (*E*)-β-caryophyllene; 3, α-humulene; 4, germacrene D by comparison of their retention times and mass spectra to those of authentic standards. *Unidentified sesquiterpenoids. Note: β-elemene was produced as a thermal rearrangement product from germacrene A in the GC injector (Additional files 4 and 5).

Next, proteins mutated at these sites were produced and analyzed. We focused on the differences between ObTPS1, a typical ECS that produced almost exclusively (*E*)-β-caryophyllene with traces of α-humulene and germacrene A, but not germacrene D, and OrTPS1, a DAS producing germacrene D and A as its two major products. These enzymes differed in the residues present at all five positively selected sites, so ObTPS1 was used as the starting point for the generation of 31 variants covering all possible intermediates in the complete conversion from these five residues to those present in OrTPS1. All 31 variants were shown to be biochemically active (Figure 4).

To better illustrate the contribution to the functional evolution from each mutant, the product profiles of all the mutants were subject to a clustering analysis based on relative abundance of the four sesquiterpene products. As shown in Figure 4, of the five single mutants, one (ObTPS1-C429G) showed a reduced proportion of (*E*)-β-caryophyllene and an elevated amount of germacrene A, but not germacrene D, while the other four displayed the wild-type phenotype. The products of three double mutants (ObTPS1-F318L + C429G, ObTPS1-C429G + V433I and ObTPS1-F318L + V433I) included germacrene D (the chromatogram of ObTPS1-F318L + V433I is shown in Figure 4 as an instance) with the first two mutants also producing significant amounts of germacrene A (Figure 4). The triple mutant ObTPS1-F318L + C429G + V433I produced comparable amounts of germacrene A, germacrene D and (*E*)-β-caryophyllene (Figure 4) and might represent the sequence of an intermediate stage gene prior to full functional divergence. Noticeably, all the proteins were grouped into two major clades. The first clade contained the proteins producing no or just minor amounts of germacrene D while the second clade contained OrTPS1 and four ObTPS1 mutants (1 triple mutant, 2 quadruple mutants and the quintuple mutant) harboring all the following switches: F318L, C429G, and V433I, further supporting the key role of these three switches in the function evolution. Finally, the quintuple mutant gave a product profile similar to that of ObTPS1-F318L + C429G + V433I rather than a true DAS (Figure 4), and thus changes at additional residue(s) are necessary for a complete functional switch from ECS to DAS.

### Biological impact of OryzaTPS1 evolution

**Figure 5:**
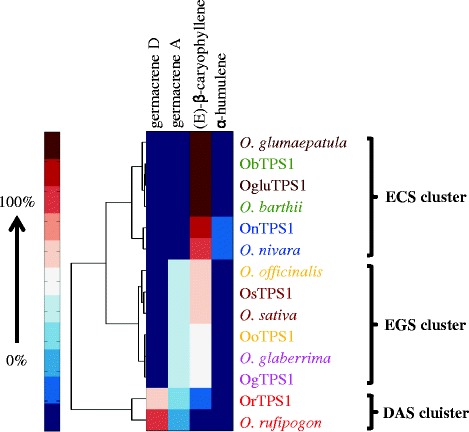
**Clustering of** ***Oryza*** **species and OryzaTPS1s.** Hierarchical cluster analysis was performed for the sesquiterpene products of seven OryzaTPS1s and the patterns of sesquiterpenes actively emitted from the seven *Oryza* species from which the corresponding OryzaTPS1s were cloned. Each enzyme clustered closely to its corresponding species showing that the products formed by *in vitro* enzyme assays were also produced by the intact plant. The name of each unique enzyme and its corresponding rice species were depicted with the same color. Relative abundance of each sesquiterpene was expressed as a percentage of the total amount of the four products. The color range represented percentages from 0% (a target compound was not detected at the expected retention time) to 100% abundance. Average values from three replicates were used.

To determine whether the evolution of the sesquiterpene synthase OryzaTPS1 is reflected in terms of the actual pattern of sesquiterpenes produced in the intact plant, we measured the volatile terpenes emitted from rice plants expressing different *OryzaTPS1s*. From our previous studies, we know that the products of OryzaTPS1 (Os08g04500) are released after herbivore damage and function together with other volatiles in attracting enemies of attacking herbivores [26,27]. A group of six rice species other than Nipponbare was subject to insect herbivory, and volatiles were sampled by headspace collection and analyzed by GC-MS. All insect-damaged rice plants emitted volatiles, including the sesquiterpene products of OryzaTPS1s (Additional file 7). Cluster analysis of the OryzaTPS1s and the rice species together was performed based on the relative abundance of the four OryzaTPS1 sesquiterpene products produced. As shown in Figure 5, this clustering led to three clades that perfectly represented the three different biochemical activities DAS, ECS and EGS. Specifically, the products of each enzyme clustered closely to the volatile products of its corresponding species. Six OryzaTPS1s, including OrTPS1, clustered immediately next to their corresponding species, while OoTPS1 belonged to the same clade as its species did. Thus, these data demonstrate that evolution of OryzaTPS1 is well correlated with changes in terpene emission profile and thus directly impacts the diversity of terpene biosynthesis of *Oryza* plants.

## Discussion

This study has demonstrated that positive Darwinian selection is an evolutionary force driving the functional divergence of terpene synthases, the pivotal enzymes for the biosynthesis of the largest class of secondary metabolites made by plants. OryzaTPS1, a terpene synthase found in rice species that produces a mixture of volatile sesquiterpenes, was subject to an overall purifying selection. This pattern is consistent with the evolution of TPS23 in maize, which may suggest that the evolution of the indirect defense traits may have been constrained in grasses. However, it was shown by phylogenetic analysis and functional characterization to have diverged from being an (*E*)-β-caryophyllene synthase (ECS) making a few other minor products to being an (*E*)-β-caryophyllene/germacrene A synthase (EGS) or a germacrene D and A synthase (DAS) (Figures 1 & 2) and molecular evolutionary analysis revealed that at least the evolution from ECS to DAS was driven by positive Darwinian selection. Functional divergence from ECS en route to DAS was partially achieved by the effects of mutations at five, particularly three, positively selected codon positions identified through the analysis using the branch-site models of the PAML package (Figures 3 & 4).

It is not particularly surprising that even a quintuple mutant of ECS in which all five target residues have been exchanged did not exhibit exact DAS activity (Figure 4). This suggests that one or more additional amino acids are involved in the activity divergence. Some of those amino acids may have been under positive selection. If so, the failure to detect the signature of positive selection could be due to the relatively small sampling size. On the other hand, the genus *Oryza* is relatively small with about 20 species; the seven species analyzed in this study account for most of the diploid *Oryza* species. Two of the five sites identified to be under positive selection exerted minimal, if any, effect on functional changes from ECS to DAS (Figure 4). One explanation for this phenomenon is epistasis, namely the function of these sites can only be manifested with the changes of additional amino acids in the appropriate context [18]. Further systemic creation and analysis of additional mutants may help clarify these possibilities.

**Figure 6:**
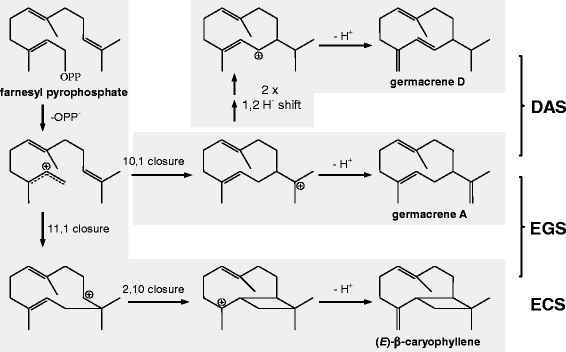
**Proposed reaction mechanism for the formation of sesquiterpene products by OryzaTPS1s.** These enzymes displayed three types of biochemical activities using farnesyl pyrophosphate as the substrate: (*E*)-β-caryophyllene synthase (ECS), (*E*)-β-caryophyllene/germacrene A synthase (EGS), and the germacrene D & germacrene A synthase (DAS). The minor product α-humulene was not considered in the reaction path.

The biochemical mechanism of functional divergence for OryzaTPS1 may arise directly from the properties of terpene synthases. While forming hundreds of monoterpene, sesquiterpene and diterpene carbon skeletons from just a few isoprenyl diphosphate substrates [5,12], terpene synthases, including OryzaTPS1, often produce multiple minor products in addition to their major products, exhibiting a type of product promiscuity attributed to their carbocationic reaction mechanisms [10]. A proposed reaction mechanism for ECS, EGS and DAS is shown in Figure 6. Common to all three enzymes is the ionization of the substrate farnesyl pyrophosphate leading to the farnesyl cation. While ECS converted this cation via a 11,1-cyclization, a subsequent 2,10-cyclization and a deprotonation to (*E*)-β-caryophyllene, EGS obtained the ability to catalyze also a 10,1-cyclization of the farnesyl cation and the conversion of the resulting germacren-11-yl cation to germacrene A. DAS, however, lost the ability to catalyze the 11,1-cyclization but is able to convert the germacren-11-yl cation via two consecutive 1,2-hydride shifts or a single 1,3-hydride shift and a subsequent deprotonation to germacrene D (Figure 6). As demonstrated by protein engineering of TPSs [33] and analysis of variants of naturally occurring TPS homologs [14,15], the proportions of major and minor products of TPSs can sometimes be readily altered by exchanges of a few amino acid residues, which is also the case for OryzaTPS1 as described here (Figure 4). These key amino acid residues are usually located within or around the synthase active site cavity [13,14]. For the functional evolution from ObTPS1 to OrTPS1, three amino acid switches F318L, C429G, and V433I as revealed by the mutagenesis analysis (using the OrTPS1 sequence as a reference, same below) did occur within the enzyme active site cavity as revealed by the structure modeling. However, two other switches, C32R and S486P, which occurred near the entrance of the active site cavity seemed not to contribute directly to enzyme functional evolution as shown by the mutagenesis analysis. This is consistent with the previous studies that the residues located within the TPS active site cavity play a more critical role in deciding the product outcome than those outside the active site cavity [13,33]. The minor activities of promiscuous terpene synthases provide the raw material for the evolution of novel enzymes in which such minor activities can become dominant ones. Functional divergence of (*E*)-β-caryophyllene/germacrene A synthase (EGS) and germacrene D and A synthase (DAS) activities from (*E*)-β-caryophyllene synthase (ECS) began with an ancestral activity that already had germacrene D and A as minor products. After functional divergence, the original function may either be retained or become lost. In the case of OryzaTPS1, the co-opted EGS and DAS activities retained the ability to produce (*E*)-β-caryophyllene, the only predominant product of the ancestral ECS, although with reduced relative abundance (Figure 1 and Additional file 3). The retention of these products may be ascribed either to natural selection or to the restricted flexibility of enzymatic mechanisms.

It should be acknowledged that the evolutionary trajectory for functional divergence of OryzaTPS1s could have occurred in a number of scenarios. If only the seven *Oryza* species studied in this paper are considered, *O. officinalis* is sister to the other six species based on the canonical *Oryza* species tree [31]. Because OoTPS1 from *O. officinalis* was an EGS, one scenario could be that the ancestral activity of the other six OryzaTPS1s is EGS. In this case, it would suggest that DAS evolved from EGS, which earlier had evolved from ECS. The functional divergence from ECS to EGS was relatively minor, affecting only the relative abundance of germacrene A (Figure 1). The five amino acid residues in ECSs and EGSs that correspond to the five sites under positive selection in DAS were highly similar, providing additional evidence on the importance of these sites on the evolution of the new activity DAS. However, it is still highly possible that DAS evolved from ECS, as inferred in this study, which would represent the ancestral activity of the six OryzaTPS1s sister to OoTPS1. In this case, the EGS activity of OoTPS1 would have evolved from an ECS after the split of *O. officinalis* from the common ancestor of the other six *Oryza* species. Functional characterization of OryzaTPS1s from additional *Oryza* species may provide better answer to this question.

The evidence for positive selection driving the diversification of secondary metabolite biosynthesis has been detected in a number of enzyme systems, such as methylthioalkylmalate synthases involved in glucosinolate biosynthesis [21] and dihydroflavonol-4-reductases involved in anthocyanin biosynthesis [24]. In all such cases, positive selection has exerted its effect on duplicated genes. In contrast, the positive selection-driven functional divergence of OryzaTPS1 in rice appears to have occurred without the direct involvement of gene duplication. In the sequenced *O. sativa* cultivar Nipponbare, *OryzaTPS1* (*Os08g04500 or OsTPS1*) and its closest paralog *Os08g07100* (50% identity at the amino acid level, Additional file 8) have been demonstrated to be derived from a duplication event that occurred before the divergence of rice (*Oryza*), maize and sorghum [28]. Os08g04500 and Os08g07100 make two completely different sets of terpene products [26]. Analysis of the whole genome sequence available for another *Oryza* species *O. glaberrima* [34] also supported that *OryzaTPS1* (gene ID ORGLA08G0019200 in this species) has not been duplicated in rice after the divergence of rice from the common ancestor of maize and sorghum as its closest paralog in this species (gene ID ORGLA08G0035300 in this species) also only possessed 48% similarity at the amino acid level to *OryzaTPS1*. Analysis of the genome sequences of other *Oryza* species analyzed in this study will be needed to provide undisputable evidence that the positive selection-driven functional divergence of OryzaTPS1 occurred without the direct involvement of gene duplication.

The divergence of OryzaTPS1 to new biochemical functions was apparent not only from *in vitro* enzyme assays (Figure 1), but also from whole plant volatile collection. There were strong correlations between the sesquiterpene profiles of individual OryzaTPS1 enzymes and the sesquiterpenes emitted from the plant from which the corresponding OryzaTPS1 was cloned (Figure 5), indicating that functional evolution of OryzaTPS1 directly impacts the phenotype of the plant. What is the biological significance of these alterations in sesquiterpene volatile emission? The volatile sesquiterpenoids emitted from insect-damaged rice and maize plants function in indirect defense as chemical cues to attract natural enemies, such as parasitic wasps [26,27,35]. Herbivore enemies have demonstrated the ability to perceive differences in terpene volatile profiles [36,37]. Therefore, the changes in rice sesquiterpene emission profiles caused by functional divergence of OryzaTPS1 may reflect the changing spectra of insect herbivores and their natural enemies present in the environments of different species. For instance, (*E*)-β-caryophyllene serves as a signal not only for the indirect defense above ground [26,28], but also for that below ground [27]. However, based on the current literature, germacrene A and germacrene D are specific signals for the indirect defense above ground [38-40]. Thus, it’s likely that evolution from ECS to DAS & EGS in certain rice lineages is driven by more serious insect challenges above ground.

The detection of positive selection as an evolutionary force driving the functional divergence of OryzaTPS1 (Figure 2) presents the first case that we are aware of in which positive selection drove the evolution of terpene synthase genes for the diversification of terpenoid biosynthesis. This study also strengthens the view that the interactions of plants with their herbivores and pathogens evolve rapidly. In the arms race with pests, plants are continually evolving novel defenses as adaptive traits under positive selection, reflected by such disparate examples as the evolution of glucosinolate biosynthetic genes [21] and plant resistance (R) genes [41] for direct defense against insect herbivores and defense against microbial pathogens, respectively. The functional divergence of the terpene synthase gene *OryzaTPS1* driven by positive selection implies the adaptive evolution of indirect defense against herbivorous insects.

## Conclusions

This study reports that positive Darwinian selection is a driving force for the functional divergence of OryzaTPS1. As the evolution of OryzaTPS1 is well correlated with changes in terpene emission profiles of *Oryza* plants, these results may imply that the sesquiterpene volatile blend produced by the *Oryza* species that contains *DAS* may be adaptive, likely in the attraction of the natural enemies of insect herbivores. This study gains us further insight into the mechanisms shaping the diversity of plant secondary metabolism.

## Methods

### Plants growth and insect treatment

Seeds of the selected *Oryza* species (Additional file 1) were obtained from the National Plant Germplasm Systems of the USDA Agricultural Research Service (http://www.ars-grin.gov/npgs/). Seeds were de-hulled and germinated at 28°C in the dark for 3 days. Seedlings were planted at eight plants per 200 ml glass jar, and grown at 28°C with 16 h of light per day. The eggs of *Spodoptera frugiperda* were purchased from Benzon Research Inc (PA, USA). Newly emerged larvae of *S. frugiperda* were transferred to 37.5 ml cups containing pinto-bean-based artificial diet as a food source in an incubator (28°C). For plant treatment, two second-instar larvae were placed on the leaves of a single 2-week-old rice seedling. After overnight (when approximately 20% of the leaf area had been consumed), insects were removed and the rice plants were subjected to volatile collection and tissue collection for RNA extraction.

### Sequence analysis

Related sequences were obtained and analyzed through the Rice Genome Annotation Project website (http://rice.plantbiology.msu.edu/index.shtml) and http://www.gramene.org/Oryza_glaberrima/Info/Index.

### Full-length cDNA cloning

Total RNA was isolated from rice tissues using Qiagen Plant RNeasy Mini Kit. cDNAs were synthesized through GE Healthcare First-Strand cDNA Synthesis Kit. Full-length cDNAs of the *TPS* genes were cloned into the vector pEXP5-CT/TOPO (Invitrogen, Carlsbad, CA, USA) and fully sequenced. The primers used for CDS cloning were: 5′- ATGGCAACCTCTGTTCCGAG-3′ (forward) and 5′-CAGTCACGCTTCATTAGAAG-3′ (reverse).

### Protein expression in *E. coli* and terpene synthase assay

An *E. coli* BL21 Codon Plus strain (Invitrogen), transformed with the appropriate expression construct (including a vector control), was used for protein expression. Expression was induced by addition of isopropyl-1-thio-d-galactopyranoside to a final concentration of 1 mm. The cells were collected by centrifugation at 4000 g for 6 min, and disrupted by a 4 × 30 sec treatment with a sonicator in chilled extraction buffer (50 mm Mopso, pH 7.0, with 5 mm MgCl_2_, 5 mm sodium ascorbate, 0.5 mm PMSF, 5 mm dithiothreitol and 10% v/v glycerol). The cell fragments were removed by centrifugation at 14 000 g, and the supernatant was desalted into assay buffer (10 mm Mopso, pH 7.0, 1 mm dithiothreitol, 10% v/v glycerol) by passage through a Econopac 10DG column (Bio-Rad). Enzyme assays were performed in a Teflon®-sealed, screw-capped 1 ml GC glass vial containing 50 μl of the bacterial extract and 50 μl assay buffer with 10 μm (*E*, *E*)-FPP, 10 mm MgCl_2_, 0.2 mm NaWO_4_ and 0.1 mm NaF. An SPME (solid phase micro-extraction) fiber consisting of 100 μm polydimethylsiloxane (Supelco) was placed into the headspace of the vial for 60 min incubation at 30°C and then inserted into the injector of the gas chromatograph for analysis of the adsorbed reaction products. Volatiles were analyzed on a Shimadzu 17A gas chromatograph coupled to a Shimadzu (http://www.shimadzu.com) QP5050A quadrupole mass selective detector. Separation was performed on a Restek SHR5XLB column (30 m × 0.25 mm internal diameter × 0.25 μm thickness, Shimadzu). Helium was used as the carrier gas (flow rate of 5 ml min^−1^), a splitless injection (injection injector temperature 250°C) was used, and a temperature gradient of 5°C min^−1^ from 40°C (3 min hold) to 240°C was applied. Products were identified by comparison of retention times and mass spectra with those of authentic reference compounds obtained from Fluka, Sigma (http://www.sigmaaldrich.com/) and W. König at the University of Hamburg.

### Plant volatile collection and identification

Volatiles emitted from insect-damaged rice plants and control rice plants were collected in an open headspace sampling system (Analytical Research Systems, Gainesville, FL, USA). Eight plants grown in a single glass jar wrapped with aluminum foil were placed in a glass chamber with a removable O-ring snap lid with an air outlet port. Charcoal-purified air entered the chamber at a flow rate of 0.8 l min^−1^ from the top through a Teflon® hose. Volatiles were collected for 4 h by pumping air from the chamber through a Super Q volatile collection trap (Analytical Research Systems). Volatiles were eluted with 40 μl of CH_2_Cl_2_, and 1-octanol was added as an internal standard for quantification. Volatile identification was conducted as described above.

### Hierarchical clustering analysis

Hierarchical clustering analysis was performed in MATLAB using the clustergram function in the bioinformatics toolbox. The Mahalanobis Distance was used to calculate the distance matrix, from which the hierarchical clusters were generated using complete linkage method. The color bar in each figure showed the relative abundance of the four sesquiterpene products (from 0% to 100%).

### Phylogeny reconstruction

To perform phylogeny reconstruction, the protein sequence alignment was performed by using the MAFFT program [42]. The phylogenetic tree based on the alignment was reconstructed using the maximum likelihood method in MEGA5 [43] with 1,000 replicates of bootstrap analysis.

### Molecular evolutionary analysis

Molecular evolution of OryzaTPS1s was analyzed using the codeml program in the PAML 4.4 package [29]. An unrooted phylogenetic tree reconstructed using the maximum likelihood method and a canonical species tree [31] containing the seven *Oryza* species included in this study were subjected to the analysis. A series of branch models were tested: the one ratio model for all the lineages and the two-ratio model for the A and D lineages respectively (labeled in the phylogenetic tree) where the original enzyme functional evolution occurred. Likelihood ratio tests (LRT) were conducted to determine which model fitted the data better. The D lineage was tested for a signature of positive selection using the branch-sites test. The positive sites with high posterior probabilities (>0.95) were obtained through Bayes Empirical Bayes analysis.

### Site-directed mutagenesis

For site-directed mutagenesis, the QuickChange site-directed mutagenesis kit (Stratagene, La Jolla, USA) was used according to the manufacturer’s instructions. Mutated genes were fully sequenced.

### Homology-based structural modeling

Protein structure modeling was performed with the SWISS-MODEL service (http://www.expasy.org/swissmod/SWISS-MODEL.html) [44] using the previously determined structure of 5-*epi*-aristolochene synthase M4 mutant ([32], PDB id, 3lz9) as the modeling template. Models were visualized and analyzed using the program PyMOL.

## Additional files

Additional file 1:
**Accessions of seven** ***Oryza*** **species analyzed and the designation of the** ***OryzaTPS1s*** **genes.**
Additional file 2
**Amino acid sequence alignment of OryzaTPS1, SbTPS4 and ZmTPS3.**
Additional file 3:
**Relative abundance of individual sesquiterpenes produced by OryzaTPS1s.**
Additional file 4:
**Chemical conversion of the sesquiterpene germacrene A to β-elemene at typical GC injector temperatures.**
Additional file 5:
**Chemical conversion of the sesquiterpene germacrene A to β-elemene at a low GC injector temperature.**
Additional file 6:
**Molecular evolutionary analysis of OryzaTPS1 using the species tree.**
Additional file 7:
**Relative abundance of individual OryzaTPS1 products emitted from insect-damaged rice plants.**
Additional file 8:
**The top five paralogs of Os08g04500 by blast search against the rice genome database with the amino acid sequence of Os08g04500 as a query.**
