# Supplementary material for: Positive Darwinian selection is a driving force for the diversification of terpenoid biosynthesis in the genus Oryza
Source: BMC Plant Biol. 2014 Sep 16;14:239. doi: 10.1186/s12870-014-0239-x (PMC4172859; doi:10.1186/s12870-014-0239-x)
Supplement: Additional file 1: — Accessions of seven Oryza species analyzed and the designation of the OryzaTPS1s genes. [file 12870_2014_239_MOESM1_ESM.pdf]

Additional file 1. Accessions of seven *Oryza* species analyzed and the designation of the *OryzaTPS1s* genes.

Species	Accession	Origin	Gene designation
O. sativa	GSOR100	Japan	*OsTPS*
O. ruffipogon	PI 590418	Myanmar	*OrTPS1*
O. nivara	PI 590425	Myanmar	*OnTPS1*
O. barthii	PI 590400	Philippines	*ObTPS1*
O. glaberrima	PI 432555	Guinea	*OgTPS1*
O. glumaepatula	PI 527368	Brazil	*OgluTPS1*
O. officinalis	PI 590412	Philippines	*OoTPS1*

Note: The second letter in the gene name is for the species name. For instance, for "*OnTPS1*", "*n*" is for the species name "*O. nivara*".
