# Supplementary material for: Positive Darwinian selection is a driving force for the diversification of terpenoid biosynthesis in the genus Oryza
Source: BMC Plant Biol. 2014 Sep 16;14:239. doi: 10.1186/s12870-014-0239-x (PMC4172859; doi:10.1186/s12870-014-0239-x)
Supplement: Additional file 2 — Amino acid sequence alignment of OryzaTPS1, SbTPS4 and ZmTPS3. [file 12870_2014_239_MOESM2_ESM.pdf]

```
OsTPS1     MATSVPSVLLLPVPTCTDMLVSPVEGGDLLHCKPHFDHHPNVWGDYFLTFSPCTPSMLLNMKRKAHVSEEQVRRMILECSSGPNL   85
OoTPS1     MATSVPSVLLLPVPTCTDMLVSPVEGGDLLHCKPHFDHHPNVWGDYFLTFSPCTPSMLLNMKRKAHVSEEQVRRMILECSSGPNL   85
OgTPS1     MATSVPSVLLLPVPTCTDMLVSPVEGGDLLHCKPHFDHHPNVWGDYFLTFSPCTPSMLLNMKRKAHVSEEQVRRMILECSSGPNL   85
OrTPS1     MATSVPSVLLLPVPTCTDMLVSPVEGGDLLHRKPHFDHHPNVWGDYFLTFSPCTPSMLLNMKRKAHVSEEQVRRMILECSSGPNL   85
ObTPS1     MATSVPSVLLLPVPTCTDMLVSPVEGGDLLHCKPHFDHHPNVWGDYFLTFSPCTPSMLLNMKRKAHVSEEQVRRMILECSSGPNL   85
OgluTPS1   MATSVPSVLLLPVPTCTDMLVSPVEGGDLLHCKPHFDHHPNVWGEYFLTFSPCTPSMLLNMKRKAHVSEEQVRRMILECSSGPNL   85
OnTPS1     MATSVPSVLLLPVPTCTNMLVSPVERGDLLHCKPHFDHHPNVWGEYFLTFSPCTPSMLLNMKREAHVTKEQVRRMILECSSGPNL   85
SbTPS4     ------------MATEAKIQIHQAQQIEDVHPK----FHSSLWGDFFLHHVPCPPVQYLMMKDKVEIMKEEVKKMLLDVDS-FDL   68
ZmTPS23    ------------MAADEARSVSRLHSEEDMHGK----HHSTLWGDFFLHHVPCRPGQYLIMKDNVEIMKEEVKKMLLDVGS-SDL   68

OsTPS1     HVKLELVDTLERLCIDYHYEKEIENVLRRVHEEEDD-TDNHYDLHTTALRFYLLRKHGYYASPDVFQRFRDEEGNFTRDDNNNGT  169
OoTPS1     HVKLELVDTLERLCIDYHYEKEIENVLRRVHEEEDD-TDNHYDLHTTALRFYLLRKHGYYASPDVFQRFRDEEGNFTCDDNNNGT  169
OgTPS1     HVKLELVDTLERLCIDYHYEKEIENVLRRVHEEEDD-TDNHYDLHTTALRFYLLRKHGYYASPDVFQRFRDEEGNFTCDDNNNGT  169
OrTPS1     HVKLELVDTLERLCIDYHYEKEIENVLRRVHEEEDD-TDNHYDLHTTALRFYLLRKHGYYASPDVFQRFRDEEGNFTCDDNNNGT  169
ObTPS1     HVKLELIDTLERLCIDYHYEKEIENVLRRVHEEEDD-TDNHYDLHTTALRFYLLRKHGYYASPDVFQRFRDEEGNFTCDDNNNGT  169
OgluTPS1   HVKLELVDTLERLCIDYHYEKEIENVLRRVHEEEDD-TDNHYDLHTTALRFYLLRKHGYHASPDVFKRFRDEEGNFTCDDNNNGT  169
OnTPS1     HIKLELIDTLERLCIDYHYEKEIDNVLRRVHEEEDDDSDNCYDLHTTALRFYLLRKHGYHASPDVFQRFRDEEGNFTCDDNNNGT  170
SbTPS4     SDKLECIDTLERLGLDYHYTKEIDKLMCNIFKASDQ----DLDLPTTSHLFYLLRKHGYHISSDVFLKFRDDKGNIVTDD----A  145
ZmTPS23    SHKLDCIDTLERLGLDYHYTKEIDELMCNVFEARDQ----DLDLTTTSQLFYLLRKHGYHISSDVFLKFRDDKGDIVTND----A  145

OsTPS1     RSMLSLYNAAHLRIHGEEILDDAIVFTRNYLQSVVKHLQSPMADEVCSALRTPLFRRPRRVEARHYISVYDKLPTRNETILEFAK  254
OoTPS1     RSMLSLYNAAHLRIHGEEILDDAIVFTRNYLQSVVKHLQSPMADEVCSALRTPLFRRPRRVEARHYISVYDKLPTRNETILEFAK  254
OgTPS1     RSMLSLYNAAHLRIHGEEILDDAIVFTRNYLQSVVKHLQSPMADEVCSALRTPLFRRPRRVEARHYISVYDKLPTRNETILEFAK  254
OrTPS1     RSMLSLYNAAHLRIHGEEILDDAIVFTRNYLQSVVKHLQSPMADEVCSALRTPLFRRPRRVEARHYISVYDKLPTRNETILEFAK  254
ObTPS1     RSMLSLYNAAHLRIHGEEILDDAIVFTRNYLQSVVKHLQSPMADEVCSALRTPLFRRPRRVEARHYISVYDKLPTRNETILEFAK  254
OgluTPS1   RSMLSLYNAAHLRIHGEEILDDAIVFTRNYLQSVVKHLQSPMADEVCSALRTPLFRRPRRVEARHYISVYDKLPTRNETILEFAK  254
OnTPS1     RSMLSLYNAAHLRIHGEEILDDAIVFTRNYLQSVVKHLQSPMTDEVCSALRTPLFRRPRRVEARHYISVYDKLPTRNETILEFAK  255
SbTPS4     RCLLLMYEAAHLRVKGEEILDNILIFTKSQLQCIVDDLEPQLKEEVKYALETPLFRRLKRVQTRQYISIYEKNTAHNNMLLEFSK  230
ZmTPS23    RCLLRMYEAAHVRVNGEEILDNILIHTKRQLQCIVDDLEPTLQEEVRYALETPLFRRLNRVQARQFISTYEKSTTRINMLLEFSK  230

OsTPS1     LDFGILQSLYCEELNILTMWWKELQLQDHLS-FARDRMVEMHFWMLGVLFEPQYSYGRTMLTKLFIFVSIFDDIYDNYSTLEESK  338
OoTPS1     LDFGILQSLYCEELNILTMWWKELQLQDHLS-FARDRMVEMHFWMLGVLFEPQYSYGRTMLTKLFIFVSIFDDIYDNYSTLEESK  338
OgTPS1     LDFGILQSLYCEELNILTMWWKELQLQDHLS-FARDRMVEMHFWMLGVLFEPQYSYGRTMLTKLFIFVSIFDDIYDNYSTLEESK  338
OrTPS1     LDFGILQSLYCEELNILTMWWKELQLQDHLS-FARDRMVEMHFWMLGVLFEPQYSYGRTMLTKLLIFVSIFDDIYDNYSTLEESK  338
ObTPS1     LDFGILQSLYCEELNILTMWWKELQLQDHLS-FARDRMVEMHFWMLGVLFEPQYSYGRTMLTKLFIFVSIFDDIYDNYSTLEESK  338
OgluTPS1   LDFGILQSLYCEELNILTMWWKELQLQDHLS-FARDRMVEMHFWMLGVLFEPQYSYGRTMLTKLFIFVSIFDDIYDNYSTLEESK  338
OnTPS1     LDFGILQSLYCEELNILTMWWKELQLQDHLS-FARDRMVEMHFWMLGVLFQPQYSYGRIMLTKLFIFVSIFDDIYDNYSTLEESK  339
SbTPS4     LDFNILLTLYCEELKDLTLWWTEFQTQANTSIYARDRMVEMHFWMMGVFFEPQYSYSRKMLTQLFMIVSILDDLYDNHCTTEEGN  315
ZmTPS23    LDFNILLTLYCEELKDLTLWWKEFQAQANTTIYARDRMVEMHFWMMGVFFEPQYSYSRKMLTQLFMIVSVLDDLYDSHCTTEEGN  315

OsTPS1     LFTEAIERWDEEAAEELPGYMKFFYKKVLTTMKSIETDLKLQGNKHVDYVKNLLIDATRCFYNEVKWRSEGADQVAATVEEHLKI  423
OoTPS1     LFTEAIERWDEEAAEELPGYMKFFYKKVLTTMKSIETDLKLQGNKHVDYVKNLLIDATRCFYNEVKWRSEGADQVAATVEEHLKI  423
OgTPS1     LFTEAIERWDEEAAEELPGYMKFFYKKVLTTMKSIETDLKLQGNKHVDYVKNLLIDATRCFYNEVKWRSEGADQVAATVEEHLKI  423
OrTPS1     LFTEAIERWDEEAAEELPGYMKFFYKKVLTTMKSIETDLKLQGNKHVDYVKNLLIDATRCYYNEVKWRSEGADQVAATVEEHLKI  423
ObTPS1     LFTEAIERWDEEAAEELPGYMKFFYKKVLTTMKSIETDLKLQGNKHVDYVKNLLIDATRCYYNEVKWRSEGADQAAATVEEHLKI  423
OgluTPS1   LFTEAIERWDEEAAEELPGYMKFFYKKVLTTMKSIETDLKLQGNKHVDYVKNLLIDATRCYYNEVKWRSEGADQAAATVEEHLKI  423
OnTPS1     LFTEAIERWDEEAAEELPGYMKFFYKKVLTTIKSIETDLKLQGNKHVDYVKNLLIDATRCYYNEVKWRSEGADQAAATVEEHLKI  424
SbTPS4     VFTAALERWDEEAVEQCPTYLRTLYVNILTTVKAIEEWLNLQNNKHAKLVKRLIIDMAKCYNAETEWRDK--KYVPATVDEHLKI  398
ZmTPS23    AFTAALQRWDEEGVEQCPTYLRTLYTNIRATIKAIEEDLNFQNNKHAKLVKGLIIDMAMCYNAETEWRDK--KYVPATVDEHLKI  398

OsTPS1     SVPSSCCMHVPVYAFVAMGNDVTTDDAINWGMAYPKIITSSCIVGRLLNDIASHEREQGSSSSSSSTVEACMREHGGITKEEAYA  508
OoTPS1     SVPSSCCMHVPVYAFVAMGNDVTTDDAINWGMAYPKIITSSCIVGRLLNDIASHEREQGSSSSSS-TVEACMREHGGITKEEAYA  507
OgTPS1     SVPSSCCMHVPVYAFVAMGNDVTTDDAINWGMAYPKIITSSCIVGRLANDIASHEREQGSSSSSS-TVEACMREHGGITKEEAYA  507
OrTPS1     SVPSSGCMHIPVYAFVAMGNDVTTDDAINWGMAYPKIITSSCIVGRLLNDIASHEREQGSSSPSS-TVEACMREHGGITKEEAYA  507
ObTPS1     SVPSSCCMHVPVYAFVAMGNDVTTDDAINWGMAYPKIITSSCIVGRLVNDIASHEREQGSSSSSS-TVEACMREHGGITKEEAYA  507
OgluTPS1   SVPSSCCMHVPVYAFVAMGNDVTADDAINWGMAYPKIITSSCIVGRLVNDIASHEREQGSS--SS-TVEACIREHGGITKEEAYA  505
OnTPS1     SVPSSCCMHIPVYAFISMGNDVTTDDAINWGIAYPKIITSSCIVGRLVNDIASHEREKGSS--SS-TVEACMREHGGITKEEAYA  506
SbTPS4     SARSSGCMHLVSQGFISMG-DVATSEAIKWASTYPKIIQAVCIIARLANDIMSYKREETSQNMVS-TVKTCAKEYG-TTVAQAIE  480
ZmTPS23    SARSSGCMHLVSQGFISMG-DVATSEALEWASTYPKIVRAVCIIARLANDIMSYKREASNNTMVS-TVQTCAKEYGTTTVEQAIE  481

OsTPS1     KLRELVEESWMDIAGECLRPAAAQ-PPPLLEAVVNATRVLDFVYKDDQDAYTHPSSLKDTIHSIYILSV-  576
OoTPS1     KLRELVEESWMDIAGECLRPAAAQ-PPPLLEAVVNATRVLDFVYKDDQDAYTHPSSLKDTIHSIYILSV-  575
OgTPS1     KLRELVEESWMDIAGECLRPAAA--PPPLLEAVVNATRVLDFVYKDDQDAYTHPSLLKDTIHSIYILSV-  574
OrTPS1     KLRELVEESWMDIAGECLRPAAAQ-PPPLLEAVVNATRVLDFVYKDDQDAYTHPSSLKDTIHSIYILSV-  575
ObTPS1     KLRELVEESWMDIAGECLRPAAAQ-PPPLLEAVVNATRVLDFLYKDDQDAYTHPSSLKDTIHSIYILSV-  575
OgluTPS1   KLRELVEESWMDIAGECLRPAAAQAAPPLLEAVVNATRVLDFLYKDDQDAYTHPSSLKDTIHSIYILPV-  574
OnTPS1     KLRELVEESWMDIAGECLRPAAAQAAPPLLEAVVNATRVLDFIYKDDQDAYTHPSSLKDTIHSIYILSV-  575
SbTPS4     KLRELIEEAWMDITEECLRQQQPK---VLLERVANLARTMDFLYKD-VDGYTDSRSIKGILDSLYVDLIN  546
ZmTPS23    KIRELIEEAWMDITHECLRQPQPK---ALLERAVNLARTMDFLYKD-ADGYTDSRSIKGILDSLYVHLID  547
```

(Additional file 2**.** Amino acid sequence alignment of OryzaTPS1s, SbTPS4 and ZmTPS3.
