# Supplementary material for: Positive Darwinian selection is a driving force for the diversification of terpenoid biosynthesis in the genus Oryza
Source: BMC Plant Biol. 2014 Sep 16;14:239. doi: 10.1186/s12870-014-0239-x (PMC4172859; doi:10.1186/s12870-014-0239-x)
Supplement: Additional file 3: — Relative abundance of individual sesquiterpenes produced by OryzaTPS1s. [file 12870_2014_239_MOESM3_ESM.pdf]

Additional file 3. Relative abundance of individual sesquiterpenes produced by OryzaTPS1s.

Category	Genes	Germacrene D	β-elemene	(*E*)-β-caryophyllene	α-humulene
**EGS**	*OsTPS1*[a]	ND[b]	37.7 ± 1.1	57.1 ± 1.8	5.2 ± 0.3
**EGS**	*OoTPS1*	ND	44.3 ± 1.4	50.3 ± 1.7	5.4 ± 0.3
**EGS**	*OgTPS1*	ND	44.5 ± 0.8	49.4 ± 0.8	6.1 ± 0.3
**ECS**	*ObTPS1*	ND	1.1 ± 0.1	96.6 ± 0.2	2.3 ± 0.1
**ECS**	*OnTPS1*	1.4 ± 0.1	2.5 ± 0.2	83.9 ± 1.0	12.2 ± 0.8
**ECS**	*OgluTPS1*	ND	0.8 ± 0.1	96.1 ± 0.3	3.1 ± 0.1
**DAS**	*OrTPS1*	55.1 ± 0.8	30.5 ± 0.6	9.5 ± 0.3	4.9 ± 0.1

[a] The total production of sesquiterpenes for each enzyme is set as 100%.
[b] ND: not detected.
