# Supplementary material for: Positive Darwinian selection is a driving force for the diversification of terpenoid biosynthesis in the genus Oryza
Source: BMC Plant Biol. 2014 Sep 16;14:239. doi: 10.1186/s12870-014-0239-x (PMC4172859; doi:10.1186/s12870-014-0239-x)
Supplement: Additional file 4: — Chemical conversion of the sesquiterpene germacrene A to β-elemene at typical GC injector temperatures. [file 12870_2014_239_MOESM4_ESM.pdf]

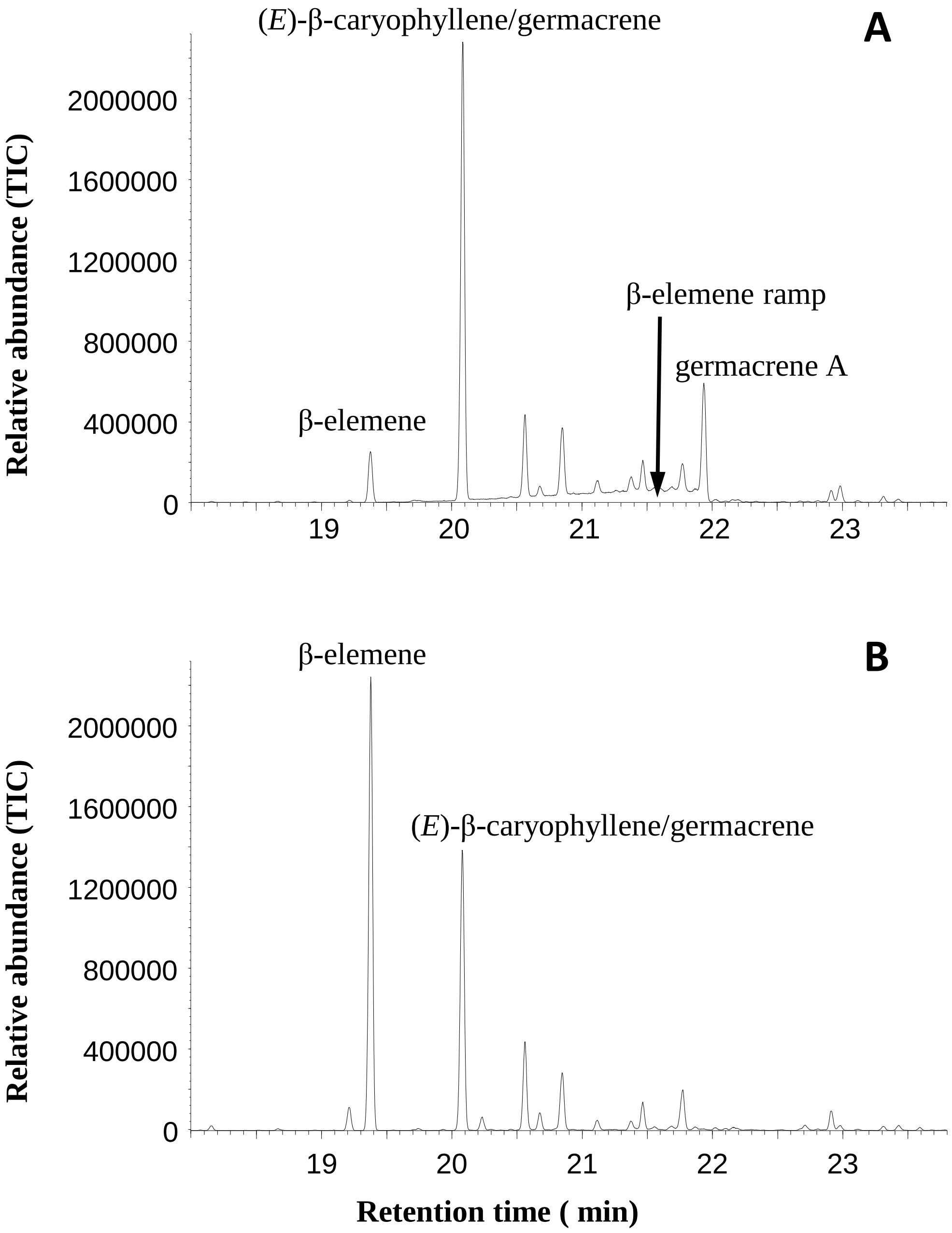

Additional file 4. Chemical conversion of the sesquiterpene germacrene A to β-elemene at typical GC injector temperatures. Chromatograms show the GC-MS analysis of terpene production for an (*E*)-β-caryophyllene/germacrene A synthase (EGS) with the GC injector temperature at (A) an atypical lower setting, 150 ℃ or (B) 250 ℃, the temperature setting usually employed for standard analysis.
