# Supplementary material for: Positive Darwinian selection is a driving force for the diversification of terpenoid biosynthesis in the genus Oryza
Source: BMC Plant Biol. 2014 Sep 16;14:239. doi: 10.1186/s12870-014-0239-x (PMC4172859; doi:10.1186/s12870-014-0239-x)
Supplement: Additional file 5: — Chemical conversion of the sesquiterpene germacrene A to β-elemene at a low GC injector temperature. [file 12870_2014_239_MOESM5_ESM.pdf]

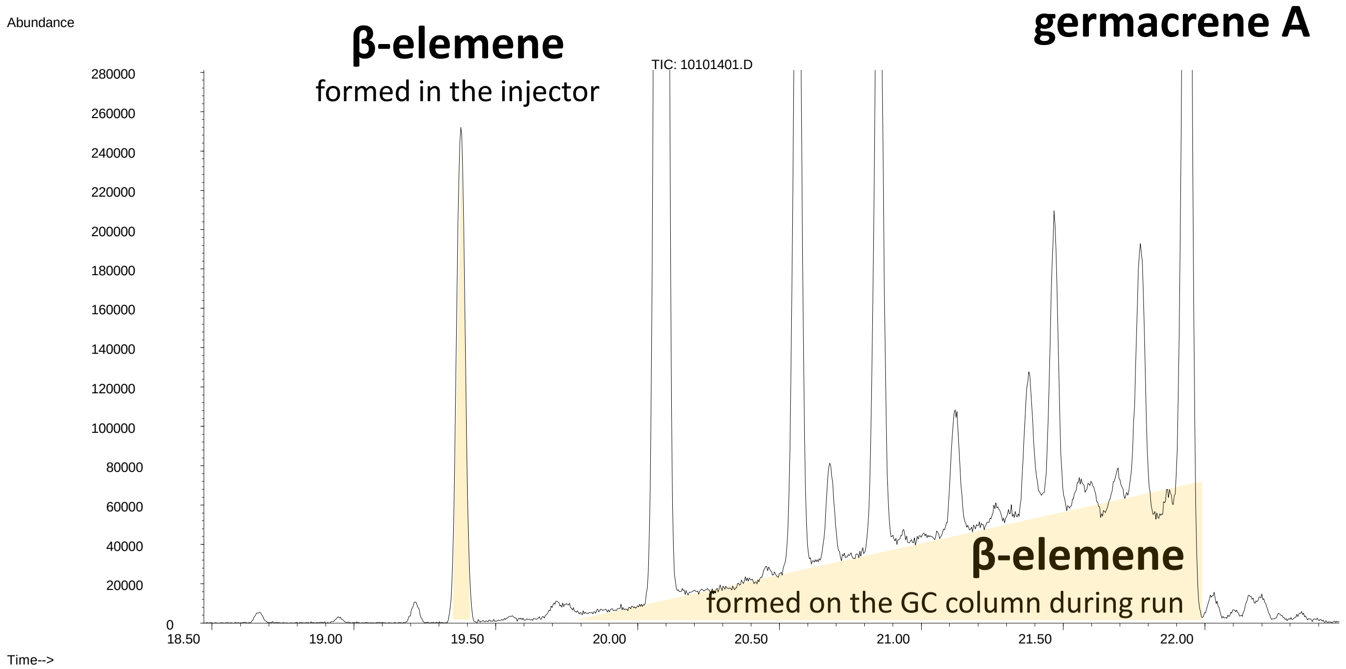

Additional file 5. Chemical conversion of the sesquiterpene germacrene A to β-elemene at a low GC injector temperature. When the injector temperature was set at 150℃, the heat-induced rearrangement of germacrene A to β-elemene occurs not only in the hot GC injector before the GC run but also during the GC run due to the increasing oven temperature. While β-elemene formed in the injector will give a sharp peak in the chromatogram, β-elemene formed during the GC run is visible as a ramp which increases with the retention time and stops exactly with the germacrene A peak. Thus the sum of the peak areas of germacrene A and β-elemene obtained with an injector temperature of 150℃ will not be the same as the peak area of β-elemene obtained with an injector temperature of 250℃.
