# Supplementary material for: Positive Darwinian selection is a driving force for the diversification of terpenoid biosynthesis in the genus Oryza
Source: BMC Plant Biol. 2014 Sep 16;14:239. doi: 10.1186/s12870-014-0239-x (PMC4172859; doi:10.1186/s12870-014-0239-x)
Supplement: Additional file 6: — Molecular evolutionary analysis of OryzaTPS1 using the species tree. [file 12870_2014_239_MOESM6_ESM.pdf]

Additional file 6. Molecular evolutionary analysis of OryzaTPS1 using the species tree

Model	Parameters estimated					LnL	Significant?	Positively selected sites[a]
Branch models								
1ω	ω_0= 0.6397					-2831.9384		
2ω_D	ω_0= 0.5732; ω_D= 999.0000					-2829.4825	Yes (P<0.05)	
Branch-site models								
Null (M1a)	ω_0= 0; ω_1= 1					-2822.1200		
Null (neutral)	Site class	0	1	2a	2b	-2822.1200		
	ω_B[b]	0	1	0	1			
	ω_A	0	1	0.4000	0.4000			
Positive selection	Site class	0	1	2a	2b	-2819.2042	Yes (vs neutral) (P<0.05)	**32R (0.977*)**<br>**318L (0.976*)**<br>**429G (0.978*)**<br>**433I (0.955*)**<br>**486P (0.977*)**
	ω_B[b]	0	1	0	1			
	ω_A	0	1	753.2913	753.2913			

- [a] the amino acids and their positions refer to those of OrTPS1M.
- [b] for Background, , ω_A for foreground(OrTPS1M);
- *denotes posterior probabilities by Bayes Empirical Bayes analysis.
