# Supplementary material for: Positive Darwinian selection is a driving force for the diversification of terpenoid biosynthesis in the genus Oryza
Source: BMC Plant Biol. 2014 Sep 16;14:239. doi: 10.1186/s12870-014-0239-x (PMC4172859; doi:10.1186/s12870-014-0239-x)
Supplement: Additional file 7: — Relative abundance of individual OryzaTPS1 products emitted from insect-damaged rice plants. [file 12870_2014_239_MOESM7_ESM.pdf]

Additional file 7. Relative abundance of individual products of OryzaTPS1 emitted from insect-damaged rice plants.

Species	Enzymes	Germacrene D	β-elemene	(*E*)-β-caryophyllene	α-humulene
O. sativa	OsTPS1	ND[b]	40.1 ± 0.6	55.4 ± 0.3	4.5 ± 0.2
O. officinalis	OoTPS1	ND	40.7 ± 0.6	59.3 ± 0.9	ND
O. glaberrima	OgTPS1	ND	43.9 ± 0.5	50.1 ± 0.9	6.0 ± 0.5
O. barthii	ObTPS1	ND	2.5 ± 0.1	95.0 ± 0.2	2.5 ± 0.1
O. nivara	OnTPS1	3.6 ± 0.1	2.8 ± 0.1	80.7 ± 0.3	12.9 ± 0.1
O. glumaepatula	OgluTPS1	ND	ND	100	ND
O. ruffipogon	OrTPS1	75.4 ± 0.5	24.6 ± 0.6	ND	ND

[a] The total emission of the four sesquiterpenes for each accession is set as 100%.
[b] ND: not detected.
