# Supplementary material for: Positive Darwinian selection is a driving force for the diversification of terpenoid biosynthesis in the genus Oryza
Source: BMC Plant Biol. 2014 Sep 16;14:239. doi: 10.1186/s12870-014-0239-x (PMC4172859; doi:10.1186/s12870-014-0239-x)
Supplement: Additional file 8: — The top five paralogs of Os08g04500 by blast search against the rice genome database with the amino acid sequence of Os08g04500 as a query. [file 12870_2014_239_MOESM8_ESM.pdf]

Additional file 8. The top five paralogs of Os08g04500 by blast search against the rice genome database with the amino acid sequence of Os08g04500 as query.

Locus	Description	Top identity
LOC_Os08g04500	terpene synthase	100%
LOC_Os08g07100	terpene synthase	50.28%
LOC_Os08g07120	putative terpene synthase	45.48%
LOC_Os08g07080	putative terpene synthase	45.33%
LOC_Os01g23530	putative terpene synthase	43.12%
LOC_Os03g24760	putative terpene synthase	40.94%
